# Estimation of radiation-induced health hazards from a “dirty bomb” attack with radiocesium under different assault and rescue conditions

**DOI:** 10.1186/s40779-021-00349-w

**Published:** 2021-12-09

**Authors:** Alexis Rump, Stefan Eder, Cornelius Hermann, Andreas Lamkowski, Patrick Ostheim, Michael Abend, Matthias Port

**Affiliations:** Bundeswehr Institute of Radiobiology, Neuherberg Str. 11, 80937 Munich, Germany

**Keywords:** Medical NRBC protection, Terrorism, Radiological emergency, Dirty bomb, Combined injuries, Triage, Acute radiation sickness, Radionuclide incorporation

## Abstract

In the case of a terrorist attack by a “dirty bomb”, blast injuries, external irradiation and the incorporation of radioactivity are to be expected. Departing from information about the radiological attack scenario with cesium-137 in the U.S. National Scenario Planning Guide, we estimated the radiological doses absorbed. Similar calculations were performed for a smaller plume size and a detonation in a subway. For conditions as described in the U.S. scenario, the committed effective dose amounted to a maximum of 848 mSv, even for very unfavorable conditions. Red bone marrow equivalent doses are insufficient to induce acute radiation sickness (ARS). In the case of a smaller plume size, the ARS threshold may be exceeded in some cases. In a subway bombing, doses are much higher and the occurrence of ARS should be expected. The health hazards from a dirty bomb attack will depend on the location and the explosive device. The derived Haddon matrix indicates that preparing for such an event includes education of all the medical staff about radiation effects, the time lines of radiation damages and the treatment priorities. Further determinants of the outcome include rapid evacuation even from difficult locations, the availability of a specific triage tool to rapidly identify victims at risk for ARS, the availability of an antidote stockpile and dedicated hospital beds to treat seriously irradiated victims.

**Supplementary Information:**

The online version contains supplementary material available at 10.1186/s40779-021-00349-w.

## Terroristic threat by dirty bombs

Risk is often defined as a product of damage size and the probability of occurrence [1]. The probability of the occurrence of a terroristic attack cannot be reasonably predicted quantitatively, so that this risk concept is not applicable. When dealing with irregular forces and applying the maxims of Clausewitz, the risk is just a function of the intention and the will to harm as well as the availability of means and abilities [2]. Therefore, assessing the risks associated with terrorism requires the identification of the potential actors and the means the terrorists may use to achieve their ends. Modes of terrorism include bombing and the use of improvised explosive devices (IED), aviation attacks and hijacking, kidnapping and assassinations, mass disruptions by attacks on critical infrastructure (e.g. energy supply, telecommunication) or the use of hazardous materials for mass destruction (CBRN terrorism) [3]. Bombs and improvised explosive devices were often used, as their components and instructions for their construction are relatively easy to obtain and only limited technical expertise is needed. The analysis of 93 reported terrorist attacks with more than 30 casualties revealed that in 88% of the cases explosions were involved [4]. A particular form of attack is suicide bombing that unlike planted IED is more precise, as the terrorist can infiltrate the target and choose the moment for the detonation. It was stated that suicide terrorism kills “about four times as many people on average than any other type of terrorism” [5]. Besides crowded urban locations, transportation systems must be viewed as one of the particularly vulnerable targets as they are difficult to protect [6, 7]. It seems untenable with present technology means to screen each commuter boarding a subway in a large city, and the emergency countermeasures in the case of a bombing incident underground are challenging. Therefore, it is not surprising that subway systems have repeatedly been selected as targets by terrorists resulting in a large number of casualties and fatalities, e.g. Tokyo (1995) or London (2005) [8, 9].

The threat of terrorists using chemical, biological or radiological materials alone or in combination with explosive devices is of great concern (CBRN terrorism). Radiological terrorism by using a radiological dispersal device (RDD, “dirty bomb”, explosive device with added radioactive material) is a significant threat, as the construction is not much more complicated than building a conventional bomb. At the difference of an improvised nuclear device (IND) using nuclear fission as a source of energy release, there is no need to master nuclear technology. Moreover, in contrast to chemicals or biologics, radionuclides are perfectly stable against the heat generated by the detonation.

Up to now, a “dirty bomb” was never detonated, but several cases point out that attacks had been planned and prepared [10]. The major challenge for the terrorist is the acquisition of the radioactive material to include into the bomb. The radioactive material may arise from an abandoned (“orphaned”) source, like in Goiania [11], it may be stolen from a facility where it is legally used or it may be bought by the terrorist pretending to be a legitimate user or as an alternative on the black market. In practice, the availability will probably be limited to radionuclide(s) used for peaceful purposes in industry, research or medicine. Radioactive sources widely available with high activities are probably of particular interest for terrorists (Table 1) [12–18]. Thus, at the end, the list of radionuclide(s) of concern that should be considered in potential scenarios of dirty bomb attacks may be quite short. In particular cesium chloride as a powder seems well suited to be pulverized into a fine dust and widely spread, so that it could be seen as the radioactive material of choice to produce an area denial effect, i.e. causing a level of contamination that would trigger area quarantine and make large-scale intensive cleanup necessary [18]. That’s probably why scenarios of radiological attacks with this radionuclide have got much attention [18, 19]. Besides the radioactive contamination of surfaces and infrastructures with a marked economic impact, the short- and long-term health effects on victims located in the proximity of the detonation point must be considered.

**Table 1:** Radioactive sources of concern for the construction of dirty bombs [12–18]

Radionuclide	Source	Activity (Ci)
**Cs-137**		
Radiation: β, ɣ	Calibration irradiator	Up to 2200
T_1/2 phys:_ 30.1 years	Blood irradiator	2000–7000 (typically 3000)
T_1/2 eff.:_ 109 days	Research irradiator	Up to 20,000
Powder, salt (CsCl)		
**Co-60**		
Radiation: β, ɣ	Teletherapy	1000–15,000
T_1/2 phys:_ 5.3 years	Gamma Knife	6000–7000
T_1/2 eff.:_ 1.6 years	Panoramic irradiator	1,000,000–7,000,000
Metal		
**Sr-90**		
Radiation: β		
T_1/2 phys:_ 28.2 years	Radioisotope thermoelectric generator	20,000–250,000
T_1/2 eff.:_ 4.6 years		
Ceramic (SrTiO_3_)		
**Ir-92**		
Radiation: β, ɣ		
T_1/2 phys:_ 73.8 days	Industrial radiography source	up to 1500
T_1/2 eff.:_ not available		
Metal		
**Pu-238**		
Radiation: α, (ɣ)	Radioisotope thermoelectric generator	up to 150,000
T_1/2 phys:_ 87.7 years		
T_1/2 eff.:_ 50 years		
Ceramic (PuO_2_)		
**Am-241**		
Radiation: α, ɣ	Well logging source	15–30
T_1/2 phys:_ 432.7 years	Smoke detectors	10^–6^
T_1/2 eff.:_ 45 years		
Pressed ceramic powder (AmO_2_)		
**Cf-252**		
Radiation: α, neutron		
T_1/2 phys:_ 2.65 years	Well logging source	2.5
T_1/2 eff.:_ 2.5 years		
Ceramic (Cf_2_O_3_)		

*Am-241*americium-241; *Cf-252* californium-252; *Co-60* cobalt-60; *Cs-137* cesium-137; *Ir-92* iridium-92; *Pu-238* plutonium-238; *Sr-90* strontium-90; *T*_*1/2 phys*_ physical half-life; *T*_*1/2 eff.*_ effective half-life

In previous studies, we focused on the medical countermeasures that should be put in place to be prepared in case of a dirty bomb attack (e.g. antidote stockpiling and screening capacities for radionuclide incorporation) and their optimal mix to maximize the benefits for the victims [20, 21]. In the present analysis, we will give an overview on the particular challenges encountered and health hazards induced in particular by radiation after a dirty bomb attack involving the same radionuclide (cesium-137), but occurring in different locations and conditions. In order to put the specific radiological issue in context, we will at first give a short overview of explosion physics and the medical challenges related to conventional terrorist bombing attacks as well as the general aspects of radiation damages.

## Physics of explosions

Chemical explosives consist of a fuel and an oxidizing component that may be included within the same molecular structure or provided by two separate compounds that are mixed [22]. The explosion is an exothermic reaction that is associated with a positive entropy change and that liberates large amounts of energy and gases in a very short time. During the decomposition of the reactants, high temperatures (3000–5000 K) and pressures (20–40 GPa, 2901 × 10^3^ − 5 802 × 10^3^ psi) are generated and the flow of the high-pressure reaction products leads to an energy transfer to the ambient non-reacted material at a high propagation velocity (several hundred m/s for the deflagration of a “low” explosive; several thousand m/s for the detonation of a “high” explosive). The front of high pressure progresses outwards, and directly behind this shock front travels the high velocity blast wind. At a defined location in the vicinity of the explosion point, the positive blast wave is immediately followed by the negative pressure or suction of the wave. The Friedlander wave is an idealized pressure wave form and in a real setting more complex profiles may be expected, in particular, if the wave is reflected on surfaces [23].

When the fireball associated with the initial high temperatures cools down, the smoke plume containing solid and gaseous particles including the decomposition products of the reaction is formed [22]. Dirt from the ground is also entrained into the fireball, and a detonation on a dirtier surface (e.g. sand, concrete) is associated with a higher amount of larger particles in a non-breathable range [24, 25]. Due to the still high temperatures, this buoyant plume is subject to a vertical draft. This vertical movement dominates the aerial transport of the particles in a first stage before the plume cools further down and particles start to be dispersed depending on the strength of the local wind [26]. The plume top height, the shape and rate of evolution will depend on the explosive load [Trinitrotoluene (TNT) equivalent explosive mass] as well as atmospheric conditions [27], and for “dirty bomb” scenarios values for the height of the plume found in the literature varies mostly from several ten meters to several hundred meters [14, 25]. Based on surveillance pictures, the shape of the particle cloud following the Oslo bombing in 2011 was shown to have had a radius and a height of about 40 m [26].

In the further course, atmospheric conditions become an increasingly dominant factor for the dimensional properties and the dispersion of the cloud and the radioactive material [27]. Important determinants are wind speed, wind direction, rainfall but also the temperature and local temperature differences as well as the time of the day (sun high in sky, low in sky or cloudy, night time). Meteorologists describe several conditions of atmospheric stability using the Pasquill–Gifford stability classes from A to F (A-D: daytime, unstable; E–F: night-time stable) [28]. For predictions, there are different types of dispersion models based on Gaussian plume models, Lagrangian puff models, particle random walk models or computational fluid dynamics [28]. In order to improve the planning and support of emergency operations in hazardous material including nuclear or radiological incidents, different software solutions have been developed, e.g. the HotSpot Health Physics codes of the National Atmospheric Release Advisory Center of the Lawrence Livermore National Laboratory [28, 29], the Hybrid Single-Particle Lagrangian Integrated Trajectory Model (HYSPLIT)[30] or the Severe Nuclear Accident Program (SNAP) of the Norwegian Meteorological Institute [31]. After the power plant accident in Chernobyl, fallout was transported northwest and north over the Baltic Sea and dispersion calculations performed in real time were very valuable in the acute phase to make predictions on the expected fallout [32].

## Injury patterns from a “dirty bomb” attack

### Blast injuries and burns

Blast injuries are divided into several categories [33]. Primary blast injuries result from the high-pressure front interacting with the human body and causing stress and shear waves in the tissues (median lethal pressure front about 50–75 psi) [34]. Gas filled organs (e.g. ears, lungs) are particularly at risk (severe lung damage at 20–30 psi) [34]. A severe blast lung will often result in immediate death, but in some patients, the development of acute respiratory distress syndrome may occur later on. Victims in the vicinity of the detonation are most endangered as the degradation of the overpressure is inversely related to the cube of the distance from the detonation point [35]. Secondary blast injuries result from projectiles/fragments/debris propelled by the blast wind, e.g. pieces of the bomb or nails intentionally added to the device (primary fragments) or small objects from the environment (secondary fragments, e.g. glass). These injuries are most common in terrorist bombings and more frequent than primary blast injuries, as the projectiles propelled often travel further than the blast wave [23]. Tertiary injuries are caused by the propulsion of the whole body on hard surfaces as the ground or propulsion of larger objects on the victim, resulting in blunt injuries. Quaternary injuries result from the liberated heat by the explosion: Burns can be classified in thermal burns caused by direct contact with the fireball and resulting in severe injuries and radiant burns (flash burns) affecting victims positioned at a greater distance from the detonation [35]. Quinary injuries may occur in the case additives like radioactive, biological or chemical materials are added to the explosive device.

The number of victims killed shows a large variability: When using nuclear weapons in Hiroshima and Nagasaki, the detonation height was chosen to maximize the effects of the pressure wave and thermal radiation (estimated yield 15 kt in Hiroshima and 21 kt in Nagasaki, detonation height 500–600 m) [36]. The fireball did not touch the surface of the earth, so delayed irradiation by fallout played only a minor role in relation to the prompt effects [36, 37]. It is estimated that in Hiroshima about 140,000 people and in Nagasaki about 70,000 people had died from the bombings by the end of 1945 [38]. However, the reported casualty figures vary greatly as the overwhelming chaos made orderly registration impossible. It is estimated that 90% of the fatalities occurred in the initial stage up to two weeks after the detonation [36]. Most of the fatalities in the first weeks died from combination injuries, but the precise contributions of blast injuries, burns and the impact of the initial radiation released are difficult to assess.

In comparison to a nuclear bombing, the death toll by the single use of a conventional explosive as in a dirty bomb attack will be much lower. The bombing attack in Beirut 1983 on a building housing 350 US Marines resulted in 346 casualties and among them 234 (68%) were immediately killed. Among the 112 survivors, only 7 victims (6.3%) died [39]. The bombing in the main railroad terminal in Bologna in 1980 resulted in 291 casualties, among them 73 (25%) immediately killed. Lethality among the hospitalized survivors was only 6% [39]. The simultaneous bombing attacks in Madrid resulted in 1800 injured and 191 fatalities (10.6%) with 177 (9%) immediately dead on the scene. Among the survivors, 775 were taken to hospital and 14 died in hospital. For the bombing attacks in London, 700 injured victims and 56 fatalities with 53 killed immediately were reported. Among the survivors, 350 were hospitalized [9].

A review of 29 terrorist bombings (8364 casualties, 903 immediate deaths) indicates that there is a dependency on the type of bombing: The immediate mortality rate amounts to 1 death among 25 victims in open air bombings, 1 among 12 in confined space bombings and 1 among 4 in bombings associated with a structural collapse [4]. In all bombing types, the analysis of injury and mortality patterns seems to confirm a biphasic mortality rate with most fatalities dying at a very early point in time and a low mortality rate in immediate survivors. This pattern seems to differ at least quantitatively from the trimodal distribution generally described for conventional blunt or penetrating trauma with 50 to 60% immediate deaths on the scene (first peak, brain injury and hemorrhage as important causes of death), 25 to 30% early death within 24 h after hospital admission (the second peak, causes like for immediate deaths, but less severe) and 10 to 20% late deaths after days to weeks (third peak, mainly due to infections and multiple organ failure, with a relative drop over time)[4, 40]. A trimodal mortality distribution has also been reported for military combat settings with however differences in the relative height of the different peaks and the causes of death in the third peak (late death mostly by central nervous system injury) compared to civilian trauma [41].

The incidence and severity of burns seem to depend on the type of bombing and to be particularly high when the detonation occurs in a confined space (on the average 22% in confined spaces vs. 1% after open air bombings) [4]. Most bombings during the terrorist wave 1994–1996 in Israel occurred in closed environments, in particular in buses. Among the 144 fatalities, 42% had severe burns with on average 32% of the total body surface affected. Among the 760 injured 12.7% had burns (mean 15% total body surface). In contrast between 2000 and 2003 explosives were mainly detonated in open areas as malls or outdoor restaurants. The major cause of death was penetrating injuries, whereas only 6.2% of the survivors had slight burns [42]. In the London bombings in 2005, severe burn injuries have been reported in particular in fatalities also suffering significant inhalation and primary blast injuries, whereas in survivors, areas not covered with clothing were mainly affected and these wounds healed within days [35].

Previous experiences seem to indicate that most victims surviving the immediate period following the explosion have a relatively good prognosis considering the mechanical injuries or burns incurred. The potential for quinary injuries, i.e. acute and long-term radiation effects in the case of a “dirty bomb”, must be thoroughly assessed in these surviving patients. In the case of combination injuries (blast injury + irradiation and/or radioactive contamination), it should be noted that mechanical trauma can cause an immediately life-threatening situation (e.g., tension pneumothorax), whereas acute radiation sickness develops with a latency ranging from days to weeks. Therefore, as in every medical emergency, the principle "treat first what kills first" applies [33, 43]. The preservation of the vital functions always has first priority and initial triage and treatment decisions must be done using the general rules of trauma care [33]. At this stage, it is not meaningful to apply triage systems specific to radiation accidents [44, 45]. These should be used later on to re-triage the patients once acute life-threatening conditions due to mechanical trauma have been treated [46, 47].

### Health hazards from irradiation

Irradiation of victims can result from radiation emanating from radionuclides suspended in the plume (irradiation by immersion, “cloud shine”) and/or from the radioactivity deposited on the ground (“ground shine”). The relative radiological doses absorbed from these two sources will depend first on the fraction of radioactivity that is aerosolized by the detonation. For ceramics, aerosolisation fractions between 2 and 40% of the contaminant mass have been reported [14, 48]. However, values might be higher for powders (cesium chloride) and lower for solid metal forms. For cobalt-60 or iridium-92 less than 1% is expected [14]. The value will also strongly depend on the construction of the explosive device.

The relative radiological doses absorbed by the “cloud shine” and “ground shine” will also vary over time as radioactive particles suspended in the plume will fall down to earth and so radioactivity deposited on the ground will be enhanced. Once all activity has been deposited, the irradiation by the “cloud shine” will be terminated and the “ground shine” will have reached its maximum. The time needed for total particle deposition on the ground will heavily depend on particle size distribution, as the deposition velocity is higher for large particles than for small particles, and different complex processes are involved (Brownian diffusion, impaction, gravitational settling, etc.) [14]. Detonation experiments have often found a particle size spectrum in the 30–100 µm range with only a small fraction of particles in the range of a few microns [49]. Simulations of “dirty bomb” scenarios have been performed assuming the percentages of breathable particles with 10–20% based on explosion dust particle size measurements [28, 50]. In the Thule incident (1968, crash in Greenland of a U.S. jet carrying nuclear bombs spreading radioactive wreckage), most particles were however of small size (only 1.3% were over 18 µm), but 80% of the radioactivity was associated with the larger particles [49, 51]. Thus, particle size and the associated radioactivity distribution may widely vary and a cautious estimation for predictions requires sensitivity analysis. Besides particle size distribution, as a second factor, the height of the plume will determine the time needed for the complete deposition on the ground.

Besides external irradiation, radioactivity may be incorporated by inhalation of contaminated air of the plume. Incorporation will also heavily depend on particle size distribution and is particularly high in the range of a few microns. Absorption rate is moreover dependent on the solubility of the material inhaled. Assuming that radioactive contaminants on the ground are not re-suspended, activity inhalation will be limited to the time particles are not still completely deposited (or the patient is evacuated). The radionuclide(s) incorporated will distribute in the body and concentrate in particular organs and tissues depending on their toxicokinetic properties. Physical decay will lead to internal irradiation with different doses absorbed by the various organs and tissues, depending on the specific affinity and accumulation site of the nuclide. Elimination will occur by a combination of physical decay and biological elimination (e.g. renal excretion) with both processes determining the effective half-life in the body [52]. Whereas external irradiation ends once the victim is evacuated from the scene, internal irradiation will go on as long as radionuclides remain in the tissues.

Irradiation even with low or moderate doses may lead to health effects in the long run [53, 54]. Radiation is associated with an increased risk of cancer mortality throughout the life of the victims depending on the absorbed dose [55]. As probability of cancer occurrence is enhanced, these damages are referred to as stochastic and it is considered that there is no threshold for this effect. Whereas the excess absolute rates for solid cancers continue to be enhanced with age, the risks of leukemia increase in the early period after irradiation to decrease thereafter [53, 56]. Besides cancers, radiation exposure has been associated with excess morbidity and mortality from non-malignant pathologies like circulatory, respiratory and digestive diseases [53, 57–59]. However, epidemiological findings on excess relative risks (ERR) show a large variability depending on the radiological doses, the age of death and confounders are difficult to control (e.g. differences between A-bomb survivors and workers in the nuclear industry) (Table 2) [53, 55, 60]. The calculated excess relative risk per unit (ERR/Gy) also depends on the dose–effect relation (linear or linear quadratic) used. Cardiovascular morbidity and mortality may be considered of particular concern [61]. Because of the high death rate from diseases of the circulatory system in the general population, even an excess relative risk that may seem small at first sight will lead to a large excess absolute risk (EAR = death rate _radiation exposed_ – death rate _non exposed_ = ERR × death rate _non exposed_) [62]. This becomes also clear when viewing the number of death cases due to cardiovascular diseases in the populations studied after radiation exposure (Table 2). Whereas ERR is suited to understand small risk differences, the EAR is more clearly related to the real burden of the disease.

**Table 2:** Comparison of excess relative risks per dose unit (ERR/Gy) with the 95% confidence interval (95% CI)

Item	Non-cancer disease	Circulatory disease	Respiratory disease	Digestive disease
Atomic bomb survivors (deceased 1966–2003) [53]				
Number of deaths	25,618	14,586	4190	2226
ERR/Gy	0.13	0.11	0.23	0.20
95% CI	(0.08–0.18)	(0.05–0.18)	(0.11–0.36)	(0.05–0.38)
Atomic bomb survivors (men exposed at the age of 20 to 60 years) [55]				
Number of deaths	4563	2571	911	370
ERR/Gy	0.12	0.16	0.04	− 0.03
95% CI	(0.01–0.24)	(0.02–0.32)	(− 0.17 to 0.30)	(− 0.35 to 0.40)
Nuclear workers (95% men) [60]				
Number of deaths	11,255	8412	792	620
ERR/Gy	0.24	0.09	1.16	0.96
95% CI	(− 0.23 to 0.78)	(− 0.43 to 0.70)	(− 0.53 to 3.84)	(< 0 to 4.52)
Nuclear workers (deceased at age < 50 years) [60]				
Number of deaths	798	516	27	82
ERR/Gy	9.10	9.36	20.35	5.67
95% CI	(2.02–19.70)	(1.64–21.50)	(< 0 to 273.00)	(< 0 to 75.00)

The radiation exposure is usually quantitated by the committed effective dose as the metric, which is defined as the total effective dose due to radionuclide incorporation absorbed over 50 years after the incorporation incident (70 years for children). The kind of radiation (alpha, beta or gamma) and the relative sensitivity of the different tissues to radiation for stochastic health effects are taken into account. The committed effective dose cannot be directly measured, but it must be calculated from the incorporated activity based on complex physiologically-oriented kinetic models in combination with a dosimetric model describing the absorption of energy in the different organs and tissues due to the radioactive decay [63]. Averaged over the genders and all age groups, the absorption of an effective dose of 1 mSv is associated with a loss of statistical lifetime of 0.4 days [64, 65]. There is no threshold level known for stochastic radiation damages (linear no-threshold model), and so for victims of a “dirty bomb” incident the total effective doses from external and internal irradiation must be taken into account for a health hazard assessment.

In the case, the committed effective dose is estimated as high (depending on the authors and guidelines > 20–200 mSv) [66], decorporation treatment should be initiated as soon as possible, provided the radionuclide is prone to such a therapy [67–69]. Two decorporation agents are of particular importance for radiological emergencies: Prussian Blue (ferric hexacyanoferrate) that is administered orally binds cesium-137 that is secreted through the bile into the gut, and thus prevents its re-absorption into the blood and enhances its elimination through the feces [67, 70, 71]. Diethylenetriaminepentaacetic acid [(Ca)DTPA and (Zn)DTPA] administered parenterally exchanges the less-firmly bound calcium-or zinc-ion for many metal radionuclides, among them plutonium-239 or americium-241, and speeds up their renal excretion [67, 72]. As some of these nuclides accumulate in “deep compartments” like the bone and then are not accessible any more to Ca (DTPA) that distributes only in the extracellular space, and moreover have a very long effective half-life in the body (e.g. plutonium 50 years, americium 45 years) [13], it is important to administer the chelator early after radioactivity incorporation, so that the nuclides can be bound as long as they are in the blood [47, 67–69]. Depending on the scale of the scenario and the therapeutic strategy, the quantitative antidote requirements and the logistic challenges may be huge [20, 21].

Besides stochastic health effects, radiation may induce deterministic damages leading to an acute radiation syndrome [73]. After irradiation, temporary prodromal symptoms appear after hours to days. The acute radiation sickness then manifests itself after a latency period from days to weeks. The higher the absorbed dose, the shorter the time to prodromal symptoms and the shorter the latency to full manifestation of the disease. Rapidly reproducing cell types are prone to cellular damages and that’s why the stem cells in the red bone marrow are particularly sensitive to ionizing radiation followed by the intestinal crypt cells [73]. The sub-syndromes of the acute radiation syndrome are shown in Table 3 [73]. There is a threshold of about 1000 mSv equivalent dose (the sensitivity factor used for the calculation of effective doses is not applied for equivalent doses!) that must be absorbed within a short timeframe to cause clinical symptoms and at first myelosuppression. Most cases observed have been caused by external irradiation, and with some exceptions (e.g. the Litvinenko case) [74], past experiences seem to indicate that radionuclide incorporation is generally not suited to deliver radiological doses to the red bone marrow within a time frame short enough to induce clinical symptoms [75]. In contrast to stochastic health effects, many hypothetical scenarios of “dirty bomb” attacks described are not associated with a risk of acute radiation sickness development [19, 28]. The total dose absorbed in 50 years is not an adequate metric to predict the occurrence of an acute radiation syndrome as the dose rates may heavily differ over time depending on the effective half-life of the radionuclide involved. In the case of cesium-137, its physical decay half-life amounts to 30 years, but the biological half-life of cesium-137 determining the effective half-life is in a range of 70 to 130 days (more precisely, the retention follows a two-exponential decay: R(*t*) = 0.1 × *e* ^(−0.347 × *t*)^ + 0.9 × *e* ^(−0.00630 × *t*)^) [76]. Therefore, 50% of the total 50 years dose will have been absorbed already within the first 3 months after incorporation (~ 1 half-life) and 94% within the first year (1 year corresponds roughly to 4 half-lives). Thus, besides the total dose, its distribution over time should be considered, and scenarios of a dirty bomb attack associated with a high equivalent dose absorbed by the red bone marrow within a short time period early after incorporation and leading to a possible occurrence of deterministic radiation effects in victims should not be fully disregarded.

**Table 3:** Whole body doses, manifestations and prognosis of the acute radiation syndrome [73]

Dose	Sub-syndrome	Clinical manifestations	Prognosis
> 1 Gy	Hematopoietic syndrome	1–2 Gy: fatigue, weakness 2–6 Gy: fever, infections, bleeding, epilation	3–4 Gy: LD_50/60_ without treatment
> 6 Gy	Gastrointestinal syndrome	High fever, diarrhea, vomiting, dizziness, disorientation, hypotension	7–8 Gy: LD_50/60_ with intensive care
> 8–10 Gy	Neurovascular syndrome	High fever, diarrhea, unconsciousness	Probable death

LD_50/60_: lethal dose in 50% of the cases within 60 days

## Examined scenarios and method of radiological dose estimation

### Scenarios of dirty bomb attacks with cesium-137

The federal interagency community in the US has developed fifteen all-hazards scenarios in order to identify the “range of response requirements” and to permit a capabilities-based planning process [19]. Moreover, the scenarios may be used as a basis for emergency response exercises. We departed from the radiological attack scenario (Scenario Nr. 11) with the given characteristics. Three dirty bombs containing each 2300 Ci (85.1 TBq) of cesium-137 are detonated almost simultaneously in three separated but regionally close cities. The explosive consists of NH_4_NO_3_ mixed with fuel oil (ANFO 95:5 by weight) and has a yield of 3000 pounds. Before the attack, the radioactive material is inserted into the explosive mixture. The detonation aerosol is lifted more than 100 feet (about 30.5 m) and contains 90% of the original cesium-137 source. The size of the particles in the plume range between 1 and 150 µm, most approximately about 100 µm. Most of the fallout drops quickly within about 500 feet (152 m) from the detonation point. The presence of radioactivity is detected by the first responders 15 min after the explosion. At each site, there are 180 fatalities, 270 injured people requiring medical care and up to 20,000 victims externally contaminated with radioactivity. In the further course, cases of acute radiation sickness do not occur.

The described scenario assumes a series of detonations and a split of the available radioactive material. We varied the scenario assuming a single detonation with a bomb containing higher cesium-137 activities up to 20,000 Ci (740 TBq) as in some research irradiators [77].

In a further simulation, we assumed that following detonation, the buoyant plume formed a smaller cloud with a radius and height of about 40 m, as described for the Oslo bombing in 2011 before further dispersion processes [26].

In addition, we considered the case of a detonation of a dirty bomb in a subway. We assumed that the dissipation of the plume is limited to the inner space of the wagon and used the size of a train of the type Siemens C2 as used in Munich (length 115 m, width 2.90 m, height 3.60 m; seats 220, space for 720 standing people) [78].

### Estimation of the radiological doses absorbed in the proximity of the detonation point

In a first assessment, we assumed that the total radioactivity contained in the bomb is dispersed in a right circular cylinder with the height corresponding to the height of the plume (30.5 m) and the base formed by a circle with the detonation point in the center and with a radius of 152 m. A cylindrical shape was used to model the initial nuclear cloud in the ARL Fallout Prediction Model and also corresponds to one of the two types of stabilized cloud shapes to model nuclear explosions by the Norwegian Meteorological Institute [30, 31, 79]. For our computations, we did however not consider a further horizontal dispersion of radioactivity by the atmospheric wind field leading to dilution over time, but only vertical movements leading to deposition of the particles on the ground, and therefore our simulations might overestimate radioactivity concentration in the plume. We also assumed that the aerosolized fraction of the activity (90%) is distributed uniformly in the volume of the cylinder immediately after detonation. The fraction of radioactivity that is not aerosolized by the explosion (10%) is considered to be immediately uniformly spread on the ground in the circular area representing the base of the cylinder. We assumed that the aerosolized part of the activity consists of a fraction of non-breathable large particles (diameter 100 µm) and a fraction of breathable small particles (5 µm) with different deposition velocities: 0.3 m/s and 0.002 m/s (smooth surfaces) or 0.4 and 0.01 m/s (sticky grass), for 100 µm and 5 µm particles respectively [80].

We calculated the time needed for the radioactive particles to completely deposit on the ground from the height of the cylinder and from the deposition velocities (*t* = *h*/*v*, separately for 100 µm and 5 µm particles), and derived the time course of the total deposited activity expressed as activity per surface unit (Ci/m^2^ or Bq/m^2^), taking also into account the fraction of radioactivity initially not aerosolized. Moreover, we computed the mean activity concentration in the volume of the cylinder (separately for 100 µm and 5 µm particles) as this is inhaled by victims in the plume as long as the particles are not fully deposited on the ground. We did not take into account that the airway (nose and mouth) of an adult is about 1.5 m above ground, as we assumed that injured victims may lie on the ground. The total inhaled activity was calculated from the mean activity concentration in the air, the respiratory time volume (3.33 × 10^–4^ m^3^/s) and the time to evacuation, or the time till complete activity deposition on the ground, in the case evacuation occurs later. Computations were performed separately for 100 µm and 5 µm particles. The geometrical figures used for the distribution volume of the radioactivity and the complete set of formulas used for calculations are displayed in the Additional file 1: Fig. S1.

The radiological doses absorbed result from internal contamination from radionuclide inhalation and external irradiation emanating from radioactivity deposited on the ground (“ground shine”) and in the plume surrounding the body (“cloud shine”) [14]. The committed effective dose for the 50 years following incorporation as well as the equivalent doses absorbed by different organs and tissues resulting from internal contamination were estimated based on the total inhaled activity using the commercial software IMBA (Integrated Modules for Bioassay Analysis) [81]. Computations were done separately for large (100 µm) and small particles (5 µm), as after inhalation deposition in the airway and absorption into the blood differ. Dose values were thereafter summed up for both particle sizes. As besides stochastic effects, we are also interested in the assessment of potential deterministic effects (induction of acute radiation sickness) resulting from acute irradiation, we determined the fraction of the total equivalent dose (50 years dose) absorbed by the red bone marrow within the first 10 days after incorporation based on the fraction of the total area under the curve (AUC) describing the activity course over time in the body (AUC till day 10 = 6.3% of total AUC)[82]. Further radionuclide bioaccumulation via ingestion was not taken into account, as we assume that after the rescue from the contaminated zone this can be prevented through official precautionary measures and the supply of uncontaminated food and drinking water can be ensured.

**Fig. 1:**
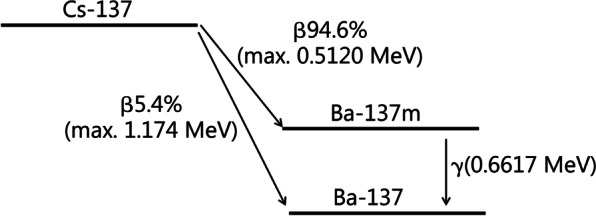
Decay of cesium-137 (Cs-137) to metastable (Ba-137 m) and stable barium-137 (Ba-137). MeV Megaelectronvolt

The radiological doses emanating from external irradiation were calculated from the external dose rate factors reported in the literature for persons standing on a radioactive surface (“ground shine”) or immersed in radioactively contaminated air (“cloud shine”) (Table 4) [83], taking into account the evacuation time and the time course of activity on the ground and in the air resulting from the deposition of the radioactive particles over time. The decay of cesium-137 is by β-decay giving rise to stable or metastable barium-137. About 94.6% of cesium-137 decays by β-emission to metastable barium-137 that further decays with a half-life of about 153 s to stable barium-137 emitting ɣ-rays (Fig. 1). This second decay is actually the reason for cesium-137 emitting ɣ-radiation. For our calculations, we merged both decay processes of cesium-137 and metastable barium-137 and for this purpose added the external dose rate factors of both nuclides. At the end, the radiological doses resulting from the different sources (internal contamination, ground and cloud shine) were summed to give a total (effective or equivalent) dose.

**Table 4:** Dose rate factors for body organs and the effective dose for immersion in contaminated air (mrem/year per µCi/m^3^) (“cloud shine”) or exposure 1 m above a contaminated ground surface (mrem/year per µCi/m^2^) (“ground shine”) [83]

Item	Effective dose	RBM	Bone	Liver	Colon	Lung	Skin
Dose rate factors for immersion in contaminated air							
Cs-137	0	0	0	0	0	0	875
Ba-137 m	3060	2740	3040	2510	2640	2680	4600
Dose rate factors for exposure above contaminated ground							
Cs-137	0	0	0	0	0	0	39.9
Ba-137 m	61.1	54.6	60.6	50.0	52.6	53.6	152

*Ba-137 m* metastable barium-137, *Cs-137* cesium-137, *RBM* red bone marrow

As absorbed radiological doses depend on many variables difficult to predict for concrete scenarios, we performed a sensitivity analysis and varied the surface characteristics of the ground (i.e. deposition velocities for large and small particles), particle size distribution and the time till the evacuation of the victims.

To simulate a situation as observed in the Oslo bombing [26], we assumed a homogeneous distribution of radioactivity in a smaller cylinder with similar height and radius of 40 m.

In order to calculate the radiological doses absorbed in a subway bombing, we assumed a homogeneous distribution of radioactivity in the volume of a cuboid calculated from the measures of a train wagon of the type Siemens C2 (length 115 m × width 2.90 m × height 3.60 m) (Additional file 2: Fig. S2) [78]. The height of the train determines the time to total deposition on the ground and maximum inhalation. Again, we considered different particle size distributions and evacuation times up to 180 min, corresponding to the time it took in the London bombing 2005 to evacuate all casualties from the subway [9]. A total evacuation time in the same order of magnitude was also reported for the Madrid bombing in 2004 (2.39 h) [9, 84].

## Results

### Open space detonation (National Planning Guide scenario 11)

**Fig. 2:**
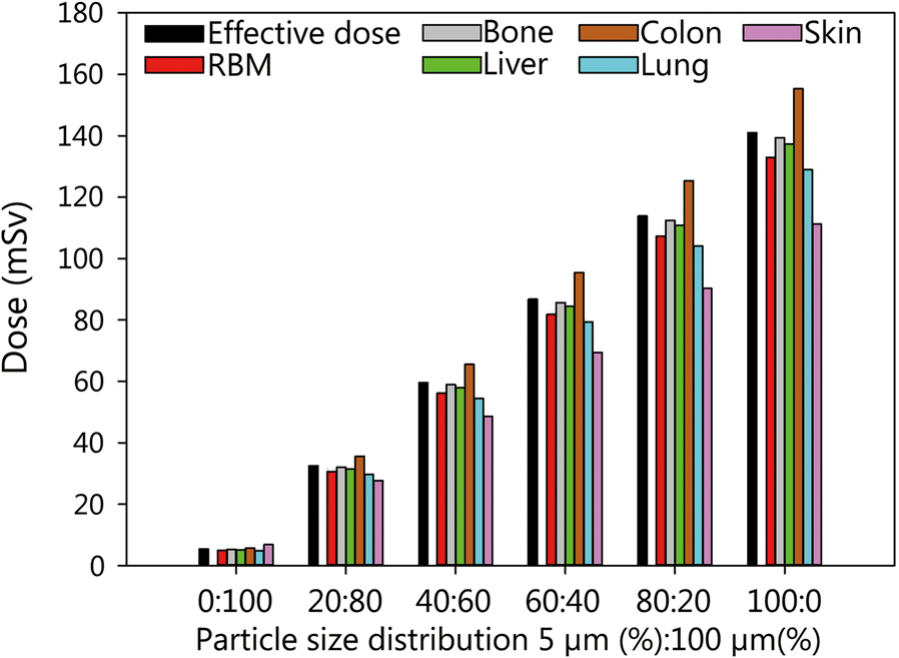
Committed effective dose (50 years) (mSv) and equivalent doses absorbed by individual organs and tissues (mSv) in a victim of a “dirty bomb” attack staying for 30 min (evacuation time) in the vicinity of the detonation point (within 150 m) depending on the distribution of particle sizes (5 µm respirable; 100 µm non-respirable). The given doses are the sum resulting from external irradiation (“ground” and “cloud shine”) and the incorporation of radioactive material by inhalation. Assumptions: activity of cesium-137 in the bomb 2300 Ci, aerosolisation of the radioactive material 90%, plume height 30.5 m (as given in the National Planning scenario Nr. 11) [19]. Assumed deposition velocity for a smooth surface: 0.3 m/s for 100 µm and 0.002 m/s for 5 µm particles [80]. RBM red bone marrow

Assuming that 2300 Ci cesium-137 is contained in the explosive device and 90% are aerosolized and the victims are evacuated from the inner zone within 30 min of the detonation, the committed effective dose will amount to 141 mSv at most (Fig. 2, Additional file 3: Table S1). The total equivalent dose absorbed by the red bone marrow over 50 years will not exceed a total of 133 mSv. Based on the fraction of the AUC under the activity-time curve, about 6.3% is absorbed within the first 10 days after the incident, i.e. the equivalent dose to the red bone marrow amounts to 8.5 mSv, so that an acute radiation syndrome is not to be expected (threshold: 1000 mSv). Differences related to the nature of the ground surface and its effect on deposition velocity are marginal from a medical point of view (smooth surface: 140.96 mSv, roughly 141 mSv; sticky grass 141.34 mSv). The radiological doses will however heavily depend on the fraction of the breathable fraction among the radioactive particles, as the internal contamination quantitatively contributes most to the total dose. The maximum dose expected results if 100% of the particles are in the breathable range (5 µm) and doses are less for lower respirable fractions (e.g. effective dose 141 mSv and 33 mSv for respirable fractions of 100% and 20%, respectively) (Fig. 2).

**Fig. 3:**
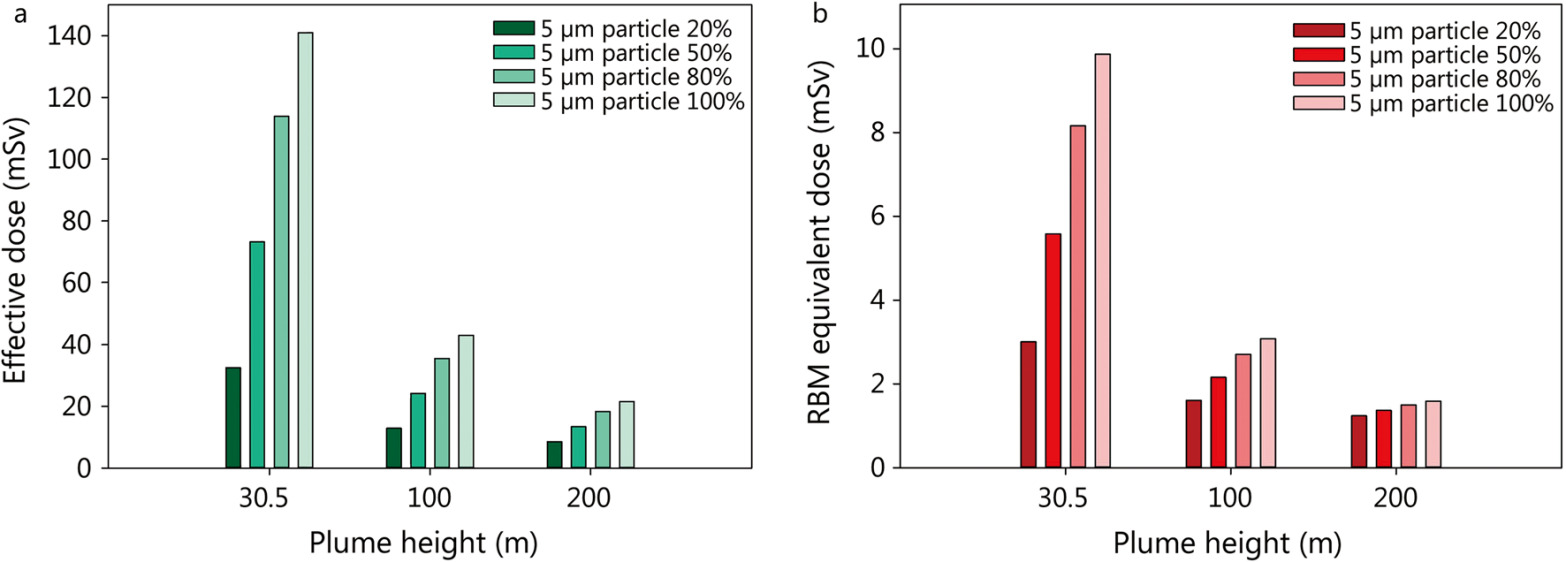
Impact of the plume height on the committed effective dose (50 years, **a**) and the equivalent dose (**b**) absorbed in the first 10 days by the red bone marrow (RBM) in a victim of a “dirty bomb” attack in the vicinity of the detonation point (within 150 m) depending on the distribution of particle sizes (5 µm respirable; 100 µm non-respirable). Assumed evacuation time: 30 min. The given doses are the sum resulting from external irradiation (“ground” and “cloud shine”) and the incorporation of radioactive material by inhalation. Assumptions: activity of cesium-137 in the bomb 2300 Ci, aerosolisation of the radioactive material 90%. Assumed deposition velocity for smooth surfaces: 0.3 m/s for 100 µm and 0.002 m/s for 5 µm particles [80]

The height of the radioactive plume is an important factor affecting the distribution volume of the aerosolized activity and in particular through its effect on the mean activity concentration in the ambient air, it affects the total absorbed radiological doses. If the plume height would rise to 100 m, instead of 30 m as described in the original scenario, the stronger activity dilution would be associated with lower doses, assuming a similar fraction of breathable particles and the same evacuation time (e.g. 43 mSv for a plume of 100 m instead 141 mSv for 30 m) (Fig. 3, Additional file 3: Table S2).

**Fig. 4:**
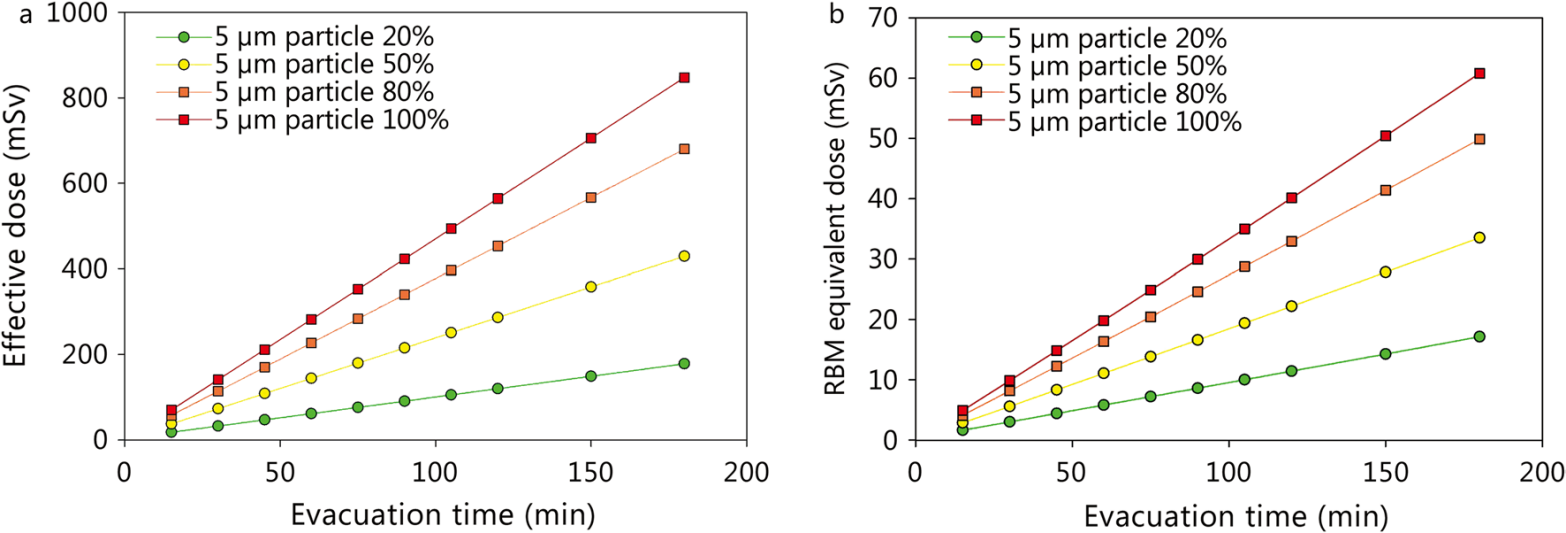
Impact of the evacuation time on the committed effective dose (50 years, **a**) and the equivalent dose (**b**) absorbed in the first 10 days by the red bone marrow (RBM) in a victim of a “dirty bomb” attack in the vicinity of the detonation point (within 150 m) depending on the distribution of particle sizes (5 µm respirable; 100 µm non-respirable). The given doses are the sum resulting from external irradiation (“ground” and “cloud shine”) and the incorporation of radioactive material by inhalation. Assumptions: Activity of cesium-137 in the bomb 2300 Ci, aerosolisation of the radioactive material 90%, plume height 30.5 m (as given in the National Planning scenario Nr. 11) [19]. Assumed deposition velocity for smooth surfaces: 0.3 m/s for 100 µm and 0.002 m/s for 5 µm particles [80]

Among the factors amenable to rescue management, the evacuation time from the zone near the detonation point impacts on the radiological doses absorbed. If evacuation is delayed up to 3 h after detonation, the committed effective doses will increase from 32.5 mSv to 178 mSv (breathable particle fraction 20%) or from 141 mSv up to 848 mSv (breathable fraction 100%). The equivalent dose absorbed by the red bone marrow within the first 10 days will nevertheless not exceed 61 mSv (breathable fraction 100%), so that even in the case of an evacuation delay of 3 h the occurrence of an acute radiation syndrome is not to be expected (Fig. 4, Additional file 3: Table S3).

**Fig. 5:**
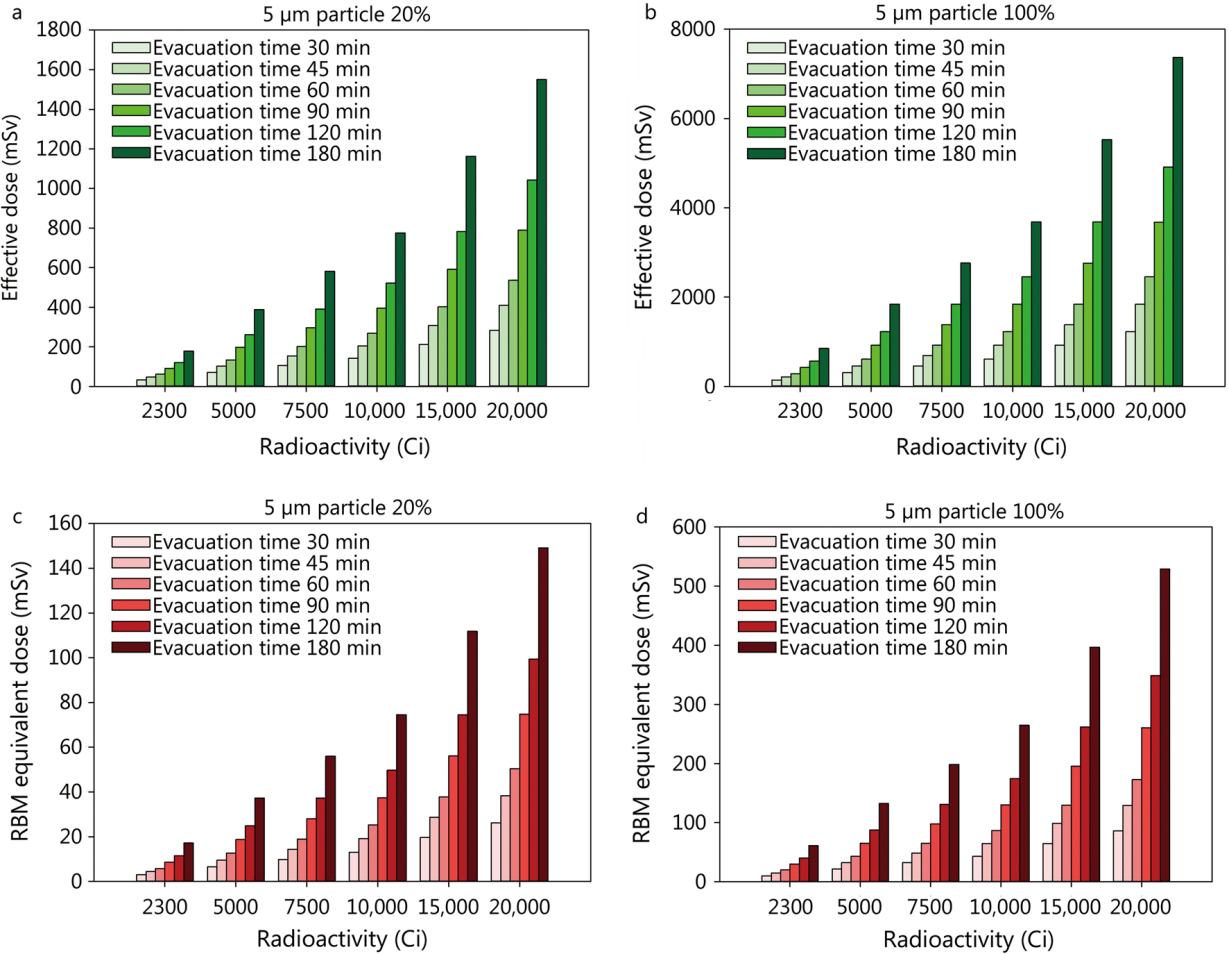
Impact of the activity of cesium-137 in the bomb on the committed effective dose (50 years) and the equivalent dose absorbed in the first 10 days by the red bone marrow (RBM) in a victim in the vicinity of the detonation point (within 150 m) depending on the distribution of particle sizes (upper figures** a** and** b**: effective dose for 20% or 100% of small 5 µm respirable particles, respectively; lower figures** c** and** d**: RBM dose for 20% or 100% of 5 µm particles, respectively) and the evacuation time. The given doses are the sum resulting from external irradiation (“ground” and “cloud shine”) and the incorporation of radioactive material by inhalation. Assumptions: aerosolisation of the radioactive material 90%, plume height 30.5 m. Assumed deposition velocity for smooth surfaces: 0.3 m/s for 100 µm and 0.002 m/s for 5 µm particles [80]

A larger radioactive load of the explosive device will expectedly be associated with higher radiological doses. Depending on the breathable particle fraction and evacuation time, the committed effective doses may reach several thousand mSv (Fig. 5, Additional file 3: Table S4). However, even if assuming a load of 20,000 Ci (content of some research irradiators), a breathable fraction of 100% and a delayed evacuation time of 3 h, the equivalent dose absorbed by the red bone marrow within 10 days will amount to 529 mSv, and thus it is below the threshold of 1000 mSv for the induction of an acute radiation syndrome (Fig. 5, Additional file 3: Table S4).

### Open space detonation (Oslo bombing plume size)

Assuming a smaller radioactive plume with a radius and height of 40 m, as described for the Oslo bombing in 2011, the activity concentration in the ambient air will be higher, leading to enhanced radiological doses. Even if the victim is evacuated within 30 min and the respirable particle fraction is 20%, the committed effective dose will amount to 373 mSv for a radioactive load of 2300 Ci and 3240 mSv for 20,000 Ci (Additional file 3: Table S5). There is an absolute indication for therapeutic decorporation by Prussian Blue. The equivalent doses absorbed during the first 10 days by the red bone marrow are nevertheless not sufficient to induce an acute radiation syndrome up to a radioactive load of 20,000 Ci, provided the victim can be evacuated from the zone at proximity of the detonation point within 30 min. Otherwise the threshold level of 1000 mSv may be exceeded, depending on the combination of radioactive load, particle size distribution and evacuation time, so that an acute radiological syndrome may occur (critical values in Table 5).

**Table 5:** Critical combinations of evacuation time, activity in the bomb and the fraction of respirable 5 µm particles (%) leading to an equivalent dose absorbed by the red bone marrow (RBM) exceeding 1000 mSv by external and internal irradiation within the first 10 days after the incident (threshold for acute radiation sickness). Assumptions: aerosolization 90%, plume size as in the Oslo bombing (radius 40 m, height 40 m) [26]

Evacuation time (min)	Activity (Ci)	5 µm particles (%)	RBM Equivalent dose (mSv)
30			< 1000
45	20,000	80	1185
	15,000	100	1069
60	20,000	50	1096
	15,000	80	1186
90	15,000	50	1231
	10,000	80	1190
	7000	100	1006
120	20,000	20	1197
	10,000	50	1096
	7000	80	1116
150	15,000	20	1121
	7500	50	1031
	5000	80	1001
180	15,000	20	1345
	7000	50	1158
	5000	80	1207

### Confined space detonation (subway attack)

**Fig. 6:**
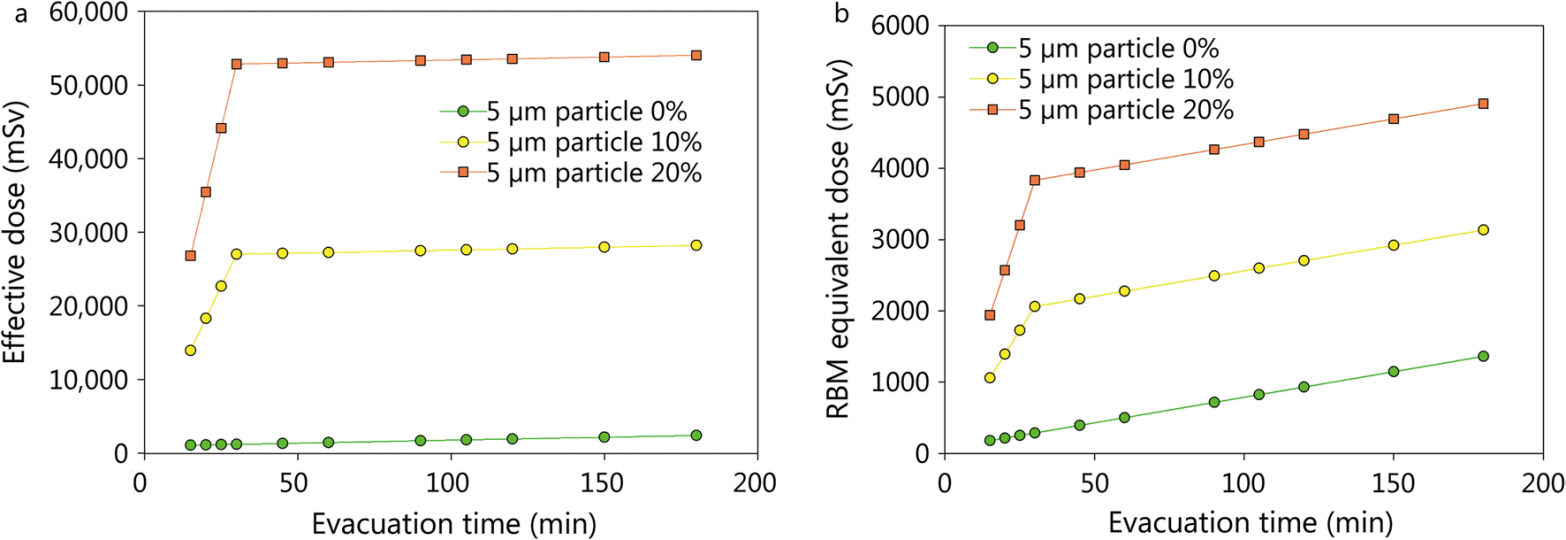
Impact of the evacuation time on the committed effective dose (50 years, **a**) and the equivalent dose (**b**) absorbed in the first 10 days by the red bone marrow (RBM) in a victim of a bombing in a confined space as a subway. The given doses are the sum resulting from external irradiation (“ground” and “cloud shine”) and the incorporation of radioactive material by inhalation. Assumptions: Activity 2300 Ci, aerosolisation of the radioactive material 90%, size of the subway wagon: length 115 m, width 2.90 m, height 3.60 m. Assumed deposition velocity for smooth surfaces: 0.3 m/s for 100 µm and 0.002 m/s for 5 µm particle [80]

Due to the limited distribution volume inside the train, the initial mean radioactivity concentration in the ambient air will be much higher after detonation than in an open space resulting in a high activity inhaled and a high “cloud shine” (assuming homogeneous distribution and 2300 Ci in the bomb, 1.72 Ci/m^3^ vs. 0.01 Ci/m^3^ in the plume modelled for the Oslo bombing). In addition, the ground surface on which activity is deposited is much smaller resulting in higher surface activities (6.90 Ci/m^2^ vs. 0.46 Ci/m^2^ on the ground for the Oslo bombing model). This leads to committed effective doses of several thousand to ten of thousand mSv (Fig. 6, Additional file 3: Table S6). Except in the case of a negligible breathable particle fraction (0% 5 µm particles, 100% 100 µm particles) and evacuation within 2 h, the threshold value of 1000 mSv equivalent dose to the red bone marrow is exceeded even in the case of very early evacuation (15 min), so that the occurrence of an acute radiation syndrome must be expected (Fig. 6, Additional file 3: Table S6).

The height of the cabin will lead even for small size particles to a shorter time to complete deposition compared to an open space detonation, limiting the time of inhalation (30 min vs. 333 min for a plume of the shape of the Oslo bombing). As even for low fractions of breathable particles (e.g. 10%) doses from internal contamination exceed doses from external irradiation, the total dose absorbed (effective dose and equivalent dose to the red bone marrow) will sharply increase up to the time point of complete activity deposition on the ground to increase at a slower pace thereafter (Fig. 6). Therefore, a very rapid evacuation is of major importance to limit health hazards by radioactivity in this case.

## Discussion and construction of a Haddon matrix

The radiological dose absorbed by victims in the proximity of the detonation point is determined primarily by factors related to the construction of the explosive device (amount of radioactivity, physicochemical properties of the compounds involved determining aerosolization and particle size, type and amount of explosives determining the height and size of the plume). The location of the detonation is of particular importance as shown by the marked differences between an open space and a confined space detonation, as it heavily affects the distribution of radioactivity. Our calculations support the common view that the detonation of a dirty bomb in an open space will rather not be suited to induce an acute radiation syndrome, although in particular circumstances it cannot be fully excluded (e.g. small plume size in combination with high activities and delayed evacuation). The medical challenge will rather be the treatment of victims with blast/mechanical trauma and taking in charge a large number of victims contaminated more or less with radioactivity with some of them needing decorporation therapy to reduce long term health effects. Identifying the patients actually needing decorporation treatment and rapidly initiating it in case of a large-scale scenario may reveal difficult. In the case of a bombing in a confined space like a subway, radiological doses should be expected to be substantially higher and in an order of magnitude suited to cause deterministic radiation injuries, i.e. acute radiation sickness, in addition to blast injuries and stochastic radiation damages.

In order to prevent an attack with a dirty bomb, or at least to reduce the probability of its occurrence, it is necessary to limit and strictly control the availability of radioactive materials and all hazardous substances and components that might be used for the construction of explosive devices. Moreover, intelligence gatherings and observation of potential perpetrators of malevolent acts are mandatory. These activities are outside the scope of the mission of rescue and medical emergency services.

After detonation, uninjured people who are able to walk should move away from the attack site for general safety reasons. In the event of radioactivity release, that will probably be unknown at this very early time point, the distance to the hypocenter is enhanced and thus external radiation intensity and the risk of contamination are reduced. An important factor with impact on the radiological dose absorbed by the victims who are unable to walk, or trapped in the area near the detonation point, is the time needed for evacuation. Besides medical reasons, fast evacuation and clearing of the target area is a must for safety reasons, as the possibility of a “second hit” must always be considered [85]. Therefore, in the case of a terrorist attack, a real “triage at the scene”, like practiced e.g. in the case of vehicle accidents with examination of the victims and categorization with different color marks, is a misperception and is not indicated. Only a distinction between dead and surviving victims should be done, and the survivors should be taken as quickly as possible to a close but safe casualty collection area where the first medical triage based on traumatic injuries should be performed [85]. Rescue and medical personnel should protect themselves from secondary contamination and incorporation [47]. Although measuring the dose rate emanating from the victims is highly advisable, the radiation intensity is probably quite low and, based on previous experiences of radiological accidents, in a case as described in the National Planning Scenario 11, at the casualty collection point, a danger for the rescue personnel from external irradiation is rather not to be expected [47, 86]. Depending on the results of the first triage, victims should be thoroughly decontaminated first or, if immediate surgery is required (e.g. massive abdominal hemorrhage after blunt trauma), taken directly to the hospital after removing clothes, i.e. removing 70–90% of the contaminating radioactivity. In that case, however, the information about incomplete decontamination is to be forwarded to the admitting facility before admission.

The time needed for the evacuation of victims will depend on the location of the bombing and the concrete situation. Evacuation from a subway system in the underground will probably be more time consuming than on the surface and will also heavily depend on the occurrence of possible structural collapses. Evacuation time is however a factor that can be influenced by mentally and materially preparing and training the rescuers and members of the medical emergency system in order to cope with this kind of disaster.

Following the first triage at the casualty collection area and a second triage at hospital admission, and after completion of urgent life-saving measures if needed, all victims should be assessed for radiation damages. For this purpose, specific triage systems based on the occurrence time of prodromal symptoms and laboratory findings (e.g. time course of blood lymphocytes) should be used. The IT-based H-module developed at the Bundeswehr Institute of Radiobiology is a modern tool for this purpose that permits to assess the probability to develop an acute radiation syndrome and thus to take a decision on an adequate patient orientation and the need of an early administration of growth factors [87]. This seems particularly important in settings where high absorbed radiation doses seem possible. In the case of an accidental setting, homogenous whole-body irradiation should not be expected and in an emergency a rapid precise dosimetric reconstruction will probably not be feasible, particularly if a large number of victims are involved. That’s why an assessment using clinical and biological parameters is of particular value.

Moreover, all victims should be considered to have potentially incorporated radioactivity with the danger of long-term health effects, although probably only a small fraction of them will actually need decorporation therapy, at least in the case of an open space bombing. The Radio-Nuclear Working Group of the WHO assumed that about 1% of potentially contaminated victims might actually require treatment [88]. The identification of this subgroup of patients is however not possible by simple medical examination, but requires technical means for activity measurement in the body. As the initiation of decorporation treatment is time critical and a delay associated with a marked loss of efficacy, according to the “urgent strategy” approach, all victims should be treated with a decorporation agent as long as a relevant incorporation of radionuclides has not been excluded by measurement [47, 67]. In an emergency setting, this is possible by mobile whole-body counters or, with a less sensitive detection limit but higher screening capacity per day, by a monitoring portal [21]. Antidote requirements may nevertheless be very high depending on the scale of the scenario [20, 21].

In the case of a bombing in a confined space with higher air activity concentrations, radionuclide incorporation by inhalation will be much more important, so that the fraction of victims actually needing decorporation treatment is expected to be higher. It was shown that the protective efficacy of Prussian Blue is limited (14 days treatment started after 6 h: reduction of the dose by 24%) and less than for example by Ca (DTPA) injection for plutonium incorporation (for similar timelines protective efficacy 42%) [68]. This is probably due to the fact that inhaled cesium-137 at first is absorbed into the blood, distributes in the body, before being secreted into the bile and intestine where it is bound by the antidote. It seems nevertheless meaningful to administer Prussian Blue generously, if stocks are available, as for short treatment periods side effects are slight, and it may prevent stochastic radiation damages, but also substantially contribute to lower the dose absorbed by the red bone marrow that according to our calculations may in some cases exceed the threshold for the induction of acute radiation sickness. At the difference of early local fallout from a nuclear detonation with doses from external irradiation by far exceeding the dangers from internal irradiation because of the large amount of very short-lived radionuclides [89], the radioactive plume from a dirty bomb detonation seems to be more comparable to regional or global fallout with the prevailing of internal contamination [37], in the cases described in this study with just 2 radioactive nuclides (cesium-137 and metastable barium-137). It is however important to note that our calculations are based on the equivalent dose absorbed by the red bone marrow within the first 10 days after the incident, a methodology previously used to assess the radiotoxicity and deterministic radiation damages of uranium at different enrichment grades [82]. This might be a quite conservative approach in order to prevent an underestimation of the absorbed dose, and using a shorter time period with the same dose rate would lead to lower absolute dose values. On the other side, extending the time frame over 10 days to calculate the dose that is compared to the acute radiation sickness threshold level (1000 mSv) cannot either be considered unjustified. Exposure time to the initial radiation of the nuclear bomb victims in Hiroshima and Nagasaki was certainly shorter. On the other side, on the occasion of the Castle Bravo nuclear test on the Marschall Islands in 1954, the residents of the Rongelap Atoll were exposed to fallout for roughly 3 days until they were evacuated [90], and the crew of the Japanese fishing boat Lucky Dragon for 14 days until they returned to Japan (with probably half the radiological dose absorbed on the first day) [91]. This more protracted irradiation over several days was nevertheless suited to induce an acute radiation syndrome in both groups of victims. Radiation exposure was in a time range comparable to the period of 10 days we used to compute the total radiological doses absorbed to appraise the possible danger of the occurrence of an acute radiation syndrome. We are not aware of an established consensus about a precise definition of what is an acute radiation exposure in a scenario like a dirty bomb attack. So, our results are very rough estimates and the order of magnitude should be used to judge the possibility of acute radiation sickness induction. Decorporation treatment initiated early must also not be considered as a guarantor to avoid acute radiation sickness, but just as a contribution to reduce red bone marrow irradiation.

The quality of care of the victims at medical facilities (hospitals or provisional hospitals) will depend on the level of preparedness for a dirty bomb attack. Education of the medical and paramedical personnel is of paramount importance, although there is no need to be a specialist in CBRN or radiobiology. Understanding the basics of health effects induced by ionizing radiation (deterministic vs. stochastic damages) and the timelines (prodromi—latency—manifestation phase of an acute radiation sickness) is a pre-requisite for a sound medical assessment and decision making. The ability to correctly identify patients who are at risk for the occurrence of an acute radiation sickness and forward them in time to specialized institutions for treatment, and the awareness that decorporation therapy should be initiated early after a suspected radioactivity incorporation, should be the goal of basic medical NR-protection education.

The great importance of the training of medical staff is impressively demonstrated by the events in Fukushima in 2011. Evacuated people and even children from the region were turned away from hospitals because it was feared they could be radioactive and pollute other people [92]. The stigmatization of irradiated patients, such as the victims of Hiroshima and Nagasaki, is a well-known, albeit regrettable social issue in Japan. Basic but solid education of health personnel on the properties and effects of radiation would certainly be the best mean to reduce unjustified fears and to ensure adequate treatment of all patients in the event of a radiological accident of any kind.

The availability of specific resources, like antidotes (growth factors and in particular decorporation agents seldom used in daily medical practice) in the required amounts and technical screening equipment for radioactivity measurement, is not the responsibility of individual medical treatment facilities, but of the authorities responsible for disaster preparedness. This requires a long-term planning and a lack of such resources cannot be reasonably compensated once the disaster has occurred.

A prerequisite for professional management of a complex and rare emergency situation is first of all the establishment of an incident command system with a fast flow of information to create a good situational awareness among the operative leadership [93]. In the case of major emergencies, local, regional and higher-level government agencies will probably all be involved, causing possible frictions and delays if the communication channels are not clearly regulated. Furthermore, the decision-making responsibilities for the release of resources must be clearly defined [94]. It can be assumed that the very special resources required in a radiological emergency are prepositioned centrally or in a few depots, so that the means of transport and the orderly distribution on the scene must be considered in advance. This is all the more important, as already mentioned, a rapid supply including uncommon antidotes (e.g. decorporation agents) in sufficient quantities is necessary to achieve maximum efficacy.

Just following the acute phase, rescue vehicles and medical facilities having transported or admitted contaminated patients will have to be screened and decontaminated quickly in order to maintain the medical services to the community at the usual level. In addition, the location of the bombing will have to be screened and decontaminated to avoid, as far as possible, disruptions of the economic activity in the area. Moreover, in the long run after a dirty bomb attack, at least a part of the victims will have to be followed up for health impairments and probably many more, victims as well as rescuers, for psychological problems.

Our calculations and the conclusions we have drawn in the discussion permit to construct a Haddon matrix. It is a tool that was initially developed in the 1970s to analyze traffic accidents and to develop preventive strategies [33]. The framework consists of three rows corresponding to the phases of the crash in time (pre-crash, crash, post-crash) and columns related to the factors that determine the severity of the consequences (host, agent/vehicle, physical environment, social environment and norms) [95, 96]. Meanwhile, this phase-factor approach has become an analysis tool applied to a variety of situations (accidents, public safety, public health, disaster planning) [97, 98], and it seems also suited to analyze terrorist attacks with a dirty bomb [99, 100]. Perspectives on a disaster may however vary, and that’s why it is very important to clearly define the event and to delineate it in time in order to separate the phases. We defined the event as the time interval from the explosion of the bomb to the evacuation of the victims from the scene and admission at a medical facility (provisional or hospital). Moreover, for a proper correct classification, it is required not to consider the time point a preparedness measure is put in place, but the phase when it becomes effective [95]. The Haddon matrix derived from our analysis is shown in Table 6.

**Table 6:** Haddon matrix applied to a dirty bomb attack

Phases	Factors			
	Host (victims)	Agent (terrorists/bombs)	Physical environment (materials/facilities)	Social environment (policies/procedures)
Pre-event (pre-bombing)	None	Gather intelligence and observe potential actors of malevolent acts Protect the access to sites storing HAZMAT or radioactive material	Ensure the availability of escape and evacuation routes from critical locations	Establish legal restrictions and control for the possession and commerce with radioactive material and components needed for an explosive device construction
Event (bombing and pre-hospital management)	Ascertain rapid evacuation from the scene Choose the right medical management priorities for treatment and transportation (“treat first what kills first”)	Screen the scene rapidly for further explosive devices (“second hit”) and other technical dangers (e.g. electricity in a subway bombing)	Ascertain the rapid availability of radiation detection devices Ascertain a sufficient number of vehicles for transportation of critical victims to hospitals as well as vehicles that may become contaminated	Educate and train EMS personnel in basic NRBC protection and medical issues related to radiation
Post-event (after evacuation to a medical care facility)	Ascertain *specific* triage to evaluate the expected health damages from irradiation and direct patients accordingly	Screen incoming people to the hospital for arms or explosive devices (to avoid “second hits” at hospital)	Ascertain a sufficient number of security personnel at emergency departments in case of panic or assaults to the personnel Ascertain the availability of sufficient antidotes and screening capacities for radioactivity as well as the logistics for distribution Ascertain the availability of a sufficient number of beds at specialized hematological wards at the regional/ national level to admit heavily irradiated victims Ascertain lines of communication from hospitals to medical NRBC experts to seek advice	Educate and train hospital staff on: 1. Self-protection 2. How to avoid secondary contamination 3. The health hazards resulting from radiation 4. Treatment priorities

*HAZMAT* hazardous materials, *EMS* emergency medical services, *NRBC* nuclear, radiological, biological and chemical

## Conclusion

Even if considering only a single radionuclide, our results show that scenarios of a dirty bomb attack may greatly differ leading to always complex but differing challenges for rescuers and emergency medical services as well as hospitals. Deterministic radiation damages will probably not be the core issue after open space detonations, but it may be a critical issue after bombing in a closed space like a subway. Offering screening for internal radioactive contamination and rapid decorporation treatment to a large number of potentially contaminated victims require adequate equipment and stockpiling as well as well functioning logistics. The Haddon matrix seems to be a well-suited instrument to analyze dirty bomb scenarios and be of help to optimize preparedness. The most important factor is probably the education of all professionals involved in coping with such a disaster (medical personnel, firefighters), as awareness and a sound judgement regarding the situation is the prerequisite for good decisions and the best use of resources.

## Supplementary Information

**Additional file 1: Fig. S1**. Formulae used to calculate the radiological doses for a cylindrical plume (open space bombing) or cubic distribution volume (confined space, subway bombing). DRF: dose rate factors for ground shine (gs) or cloud shine (immersion, cs) for the effective dose or the red bone marrow equivalent dose. Tdep: time until complete particle deposition on the ground; Tevac: evacuation time from the scene. Calculations are done for 5 µm and 100 µm particles (differences in the sedimentation velocities) and thereafter summed up to the total dose.

**Additional file 2: Fig. S2**. Exterior and interior view of a Siemens C2 subway train as used for our calculations.

**Additional file 3: Table S1**. Committed effective dose and total equivalent doses absorbed over 50 years by organs in a victim of a “dirty bomb” attack staying for 30 min in the vicinity of the detonation point depending on the distribution of particle sizes (5 µm or 100 µm). **Table S2**. Committed effective dose and equivalent dose absorbed in the first 10 days (difference to values in Table S1) by the red bone marrow (RBM) in a victim of a “dirty bomb” attack in the vicinity of the detonation point depending on the height of the plume, the distribution of particle sizes (5 µm or 100 µm) and the evacuation time. **Table S3**. Impact of the evacuation time on the committed effective dose and equivalent dose absorbed in the first 10 days by the red bone marrow (RBM) in a victim of a “dirty bomb” attack in the vicinity of the detonation point depending on the distribution of particle sizes (5 µm or 100 µm). **Table S4**. Committed effective dose and equivalent dose absorbed in the first 10 days by the red bone marrow (RBM) in a victim of a “dirty bomb” attack in the vicinity of the detonation point depending on the activity in the bomb, the distribution of particle sizes (5 µm or 100 µm) and the evacuation time. **Table S5**. Committed effective dose and equivalent dose absorbed in the first 10 days by the red bone marrow (RBM) in a victim of a “dirty bomb” attack in the vicinity of the detonation point depending on the activity of the bomb, the distribution of particle sizes (5 µm or 100 µm) and the evacuation time. **Table S6**. Committed effective dose and equivalent dose absorbed within the first 10 days by the red bone marrow (RBM) in a victim of a “dirty bomb” attack in a subway depending on the evacuation time and the distribution of particle sizes (5 µm or 100 µm).

## Data Availability

Not applicable. All calculations are based on data previously published in technical compilations, original articles or reviews cited in the text.

## Abbreviations

AUC: Area under the curve
Ba-137: Barium-137
Ba-137m: Metastable barium-137
Ca (DTPA): Calcium (diethylenetriaminepentaacetic acid)
CBRN: Chemical, Biological, Radiological, Nuclear
Cs-137: Cesium-137
CsCl: Cesium chloride
EAR: Excess absolute risk
EMS: Emergency medical services
ERR: Excess relative risk
Gpa: Gigapascal (10^9^ Pascal)
Gy: Gray
HAZMAT: Hazardous materials
HYSPLIT: Hybrid Single-Particle Lagrangian Integrated Trajectory Model
IED: Improvised explosive device
IMBA: Integrated Modules for Bioassay Analysis
IND: Improvised Nuclear Device
K: Kelvin
MeV: Megaelectronvolt (10^6^ Electronvolt)
mSv: Millisievert (10^–^^3^ Sievert)
Psi: Pound-force per square inch
RDD: Radiological Dispersal Device
SNAP: Severe Nuclear Accident Program
TBq: Terabecquerel (10^12^ Becquerel)
TNT: Trinitrotoluene

## Glossary

Absorbed dose: The energy deposited in a target by radiation. Unit: Gray (Gy). 1 Gy = 1 J/kg
Activity: The decay rate of a radionuclide. Unit: Becquerel (Bq) or Curie (Ci). 1 Bq = 1 radioactive decay per second. 1 mCi = 37 MBq
Committed effective dose: The total effective dose absorbed over 50 years (for an adult) after the incorporation of radionuclide(s). Unit: Sievert (Sv)
Deterministic health effects: Effects that occur if a threshold dose is exceeded. The symptoms and the severity of the effects depend on the dose level. Example: the acute radiation syndrome
Equivalent dose: The absorbed dose multiplied by a factor that takes into account the biological effectiveness of the type of radiation (e.g. alpha-, beta- or gamma-radiation). Unit: Sievert (Sv)
Effective dose: The sum of the equivalent doses absorbed by the organs and tissues multiplied by tissue weighting factors reflecting the sensitivity to radiation. The effective dose reflects the stochastic health risks. Unit: Sievert (Sv)
Radionuclide: A nuclide is an atom characterized by the number of protons and neutrons. A radionuclide is an unstable nuclide emitting ionizing radiation
Stochastic health effects: Effects whose probability to occur depends on the dose (e.g. occurrence of cancer after radiation exposure). However, the level of the dose does not determine the severity of the effects/symptoms. There is no threshold level
