# Supplementary figures and images for: Estimation of radiation-induced health hazards from a “dirty bomb” attack with radiocesium under different assault and rescue conditions
Source: Mil Med Res. 2021 Dec 9;8:65. doi: 10.1186/s40779-021-00349-w (PMC8656004; doi:10.1186/s40779-021-00349-w)

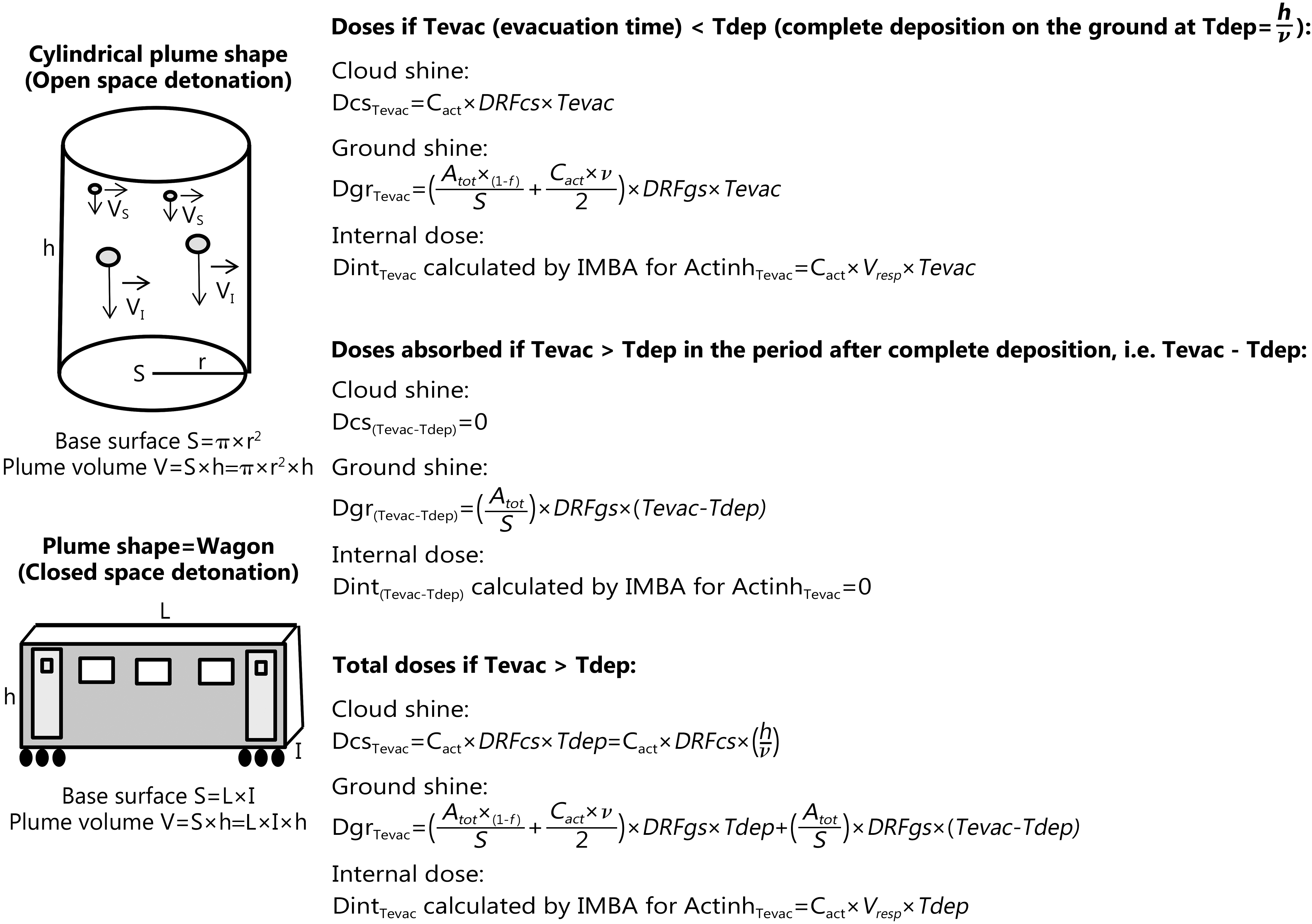

Supplement: Supplementary file 1 — Additional file 1: Fig. S1. Formulae used to calculate the radiological doses for a cylindrical plume (open space bombing) or cubic distribution volume (confined space, subway bombing). DRF: dose rate factors for ground shine (gs) or cloud shine (immersion, cs) for the effective dose or the red bone marrow equivalent dose. Tdep: time until complete particle deposition on the ground; Tevac: evacuation time from the scene. Calculations are done for 5 µm and 100 µm particles (differences in the sedimentation velocities) and thereafter summed up to the total dose. [file 40779_2021_349_MOESM1_ESM.tif]

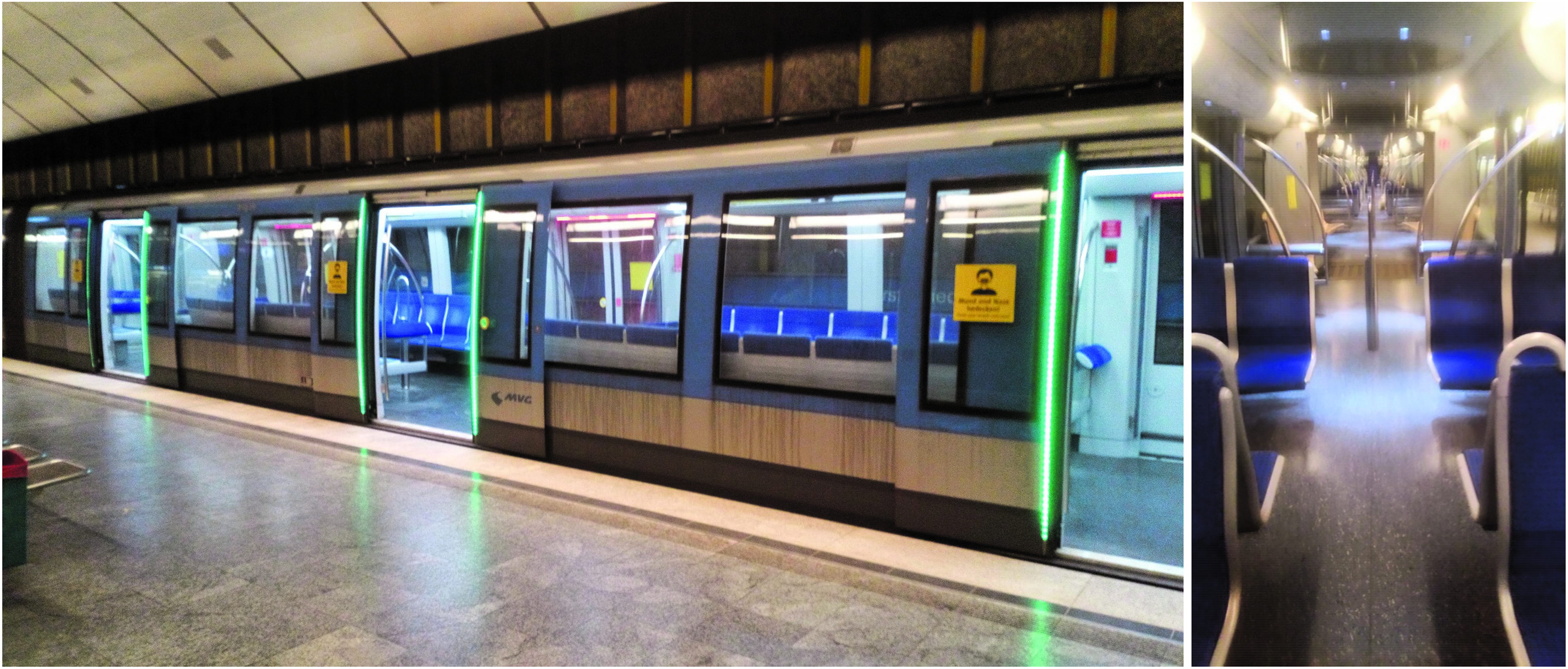

Supplement: Supplementary file 2 — Additional file 2: Fig. S2. Exterior and interior view of a Siemens C2 subway train as used for our calculations. [file 40779_2021_349_MOESM2_ESM.tif]
