# Supplementary material for: Estimation of radiation-induced health hazards from a “dirty bomb” attack with radiocesium under different assault and rescue conditions
Source: Mil Med Res. 2021 Dec 9;8:65. doi: 10.1186/s40779-021-00349-w (PMC8656004; doi:10.1186/s40779-021-00349-w)
Supplement: Supplementary file 3 — Additional file 3: Table S1. Committed effective dose and total equivalent doses absorbed over 50 years by organs in a victim of a “dirty bomb” attack staying for 30 min in the vicinity of the detonation point depending on the distribution of particle sizes (5 µm or 100 µm). Table S2. Committed effective dose and equivalent dose absorbed in the first 10 days (difference to values in Table S1) by the red bone marrow (RBM) in a victim of a “dirty bomb” attack in the vicinity of the detonation point depending on the height of the plume, the distribution of particle sizes (5 µm or 100 µm) and the evacuation time. Table S3. Impact of the evacuation time on the committed effective dose and equivalent dose absorbed in the first 10 days by the red bone marrow (RBM) in a victim of a “dirty bomb” attack in the vicinity of the detonation point depending on the distribution of particle sizes (5 µm or 100 µm). Table S4. Committed effective dose and equivalent dose absorbed in the first 10 days by the red bone marrow (RBM) in a victim of a “dirty bomb” attack in the vicinity of the detonation point depending on the activity in the bomb, the distribution of particle sizes (5 µm or 100 µm) and the evacuation time. Table S5. Committed effective dose and equivalent dose absorbed in the first 10 days by the red bone marrow (RBM) in a victim of a “dirty bomb” attack in the vicinity of the detonation point depending on the activity of the bomb, the distribution of particle sizes (5 µm or 100 µm) and the evacuation time. Table S6. Committed effective dose and equivalent dose absorbed within the first 10 days by the red bone marrow (RBM) in a victim of a “dirty bomb” attack in a subway depending on the evacuation time and the distribution of particle sizes (5 µm or 100 µm). [file 40779_2021_349_MOESM3_ESM.pdf]

Table S1 Committed effective dose and total equivalent doses absorbed over 50 years by organs in a victim of a “dirty bomb” attack staying for 30 min in the vicinity of the detonation point depending on the distribution of particle sizes (5 µm or 100 µm)

Particle size 5 µm (%)	Particle size 100 µm (%)	Effective dose (mSv)	RBM (mSv)	Bone (mSv)	Liver (mSv)	Colon (mSv)	Lung (mSv)	Skin (mSv)
0	100	5.41	5.00	5.30	5.07	5.65	4.86	6.81
20	80	32.52	30.58	32.10	31.51	35.58	29.68	27.69
40	60	59.63	56.17	58.89	57.95	65.51	54.50	48.57
50	50	73.18	68.96	72.29	71.17	80.48	66.90	59.00
60	40	86.74	81.75	85.68	84.39	95.44	79.31	69.44
80	20	113.85	107.33	112.47	110.83	125.37	104.13	90.32
100	0	140.96	132.92	139.27	137.27	155.30	128.95	111.19

Assumptions: Cesium-137 activity 2300 Ci, aerosolisation 90%, plume height 30.5 m, deposition velocity 0.3 m/s for 100 µm and 0.002 m/s for 5 µm particles. *RBM* red bone marrow

Table S2 Committed effective dose and equivalent dose absorbed in the first 10 days (difference to values in Table S1) by the red bone marrow (RBM) in a victim of a “dirty bomb” attack in the vicinity of the detonation point depending on the height of the plume, the distribution of particle sizes (5 µm or 100 µm) and the evacuation time

Evacuation time (min)	Fraction of respirable particles 5 µm (%)							
	20	50	80	100	20	50	80	100
	Effective dose (50 years) (mSv)				RBM Equivalent dose (10 days) (mSv)			
Plume height 30.5 m								
30	32.52	73.18	113.85	140.96	3.01	5.58	8.16	9.87
45	47.07	108.72	170.38	211.49	4.40	8.32	12.24	14.85
Plume height 100 m								
30	12.88	24.19	35.5	43.04	1.61	2.16	2.71	3.08
45	17.63	35.24	52.84	64.58	2.31	3.18	4.05	4.63
Plume height 200 m								
30	8.52	13.41	18.31	21.58	1.24	1.37	1.50	1.59
45	11.12	19.09	27.06	32.37	1.80	2.02	2.24	2.39

Assumptions: cesium-137 activity 2300 Ci, aerosolisation 90%, deposition velocity 0.3 m/s for 100 µm and 0.002 m/s for 5 µm particles.

Table S3 Impact of the evacuation time on the committed effective dose and equivalent dose absorbed in the first 10 days by the red bone marrow (RBM) in a victim of a "dirty bomb" attack in the vicinity of the detonation point depending on the distribution of particle sizes (5 µm or 100 µm)

Evacuation time (min)	Fraction of respirable particles 5 µm (%)							
	20	50	80	100	20	50	80	100
	Effective dose (50 years) (mSv)				RBM Equivalent dose (10 days) (mSv)			
15	17.98	37.66	57.34	70.47	1.63	2.86	4.10	4.92
30	32.52	73.18	113.85	140.96	3.01	5.58	8.16	9.87
45	47.07	108.72	170.38	211.49	4.40	8.32	12.24	14.85
60	61.62	144.28	226.94	282.04	5.80	11.07	16.34	19.85
75	76.18	179.85	283.51	352.63	7.20	13.83	20.46	24.88
90	90.74	215.43	340.11	423.24	8.60	16.60	24.60	29.94
105	105.31	251.03	396.74	493.88	10.02	19.39	28.77	35.02
120	119.89	286.64	453.39	564.55	11.43	22.19	32.95	40.13
150	149.06	357.91	566.75	705.99	14.28	27.83	41.39	50.42
180	178.26	429.24	680.21	847.53	17.15	33.53	49.90	60.82

Assumptions: cesium-137 activity 2300 Ci, aerosolisation 90%, plume height 30.5 m, deposition velocity 0.3 m/s for 100 µm and 0.002 m/s for 5 µm particles.

Table S4 Committed effective dose and equivalent dose absorbed in the first 10 days by the red bone marrow (RBM) in a victim of a "dirty bomb" attack in the vicinity of the detonation point depending on the activity in the bomb, the distribution of particle sizes (5 μm or 100 μm) and the evacuation time

Activity (Ci)	Fraction of respirable particles 5 μm (%)			
	20	100	20	100
	Effective dose (50 years) (mSv)		RBM Equivalent dose (10 days) (mSv)	
Evacuation time 30 min				
2300	32.52	140.96	3.01	9.87
5000	70.69	306.44	6.54	21.47
7000	98,97	429.01	9.16	30.05
7500	106,04	459.66	9.82	32.20
10,000	141,38	612.88	13.09	42.93
15,000	212.07	919.32	19.63	64.40
20,000	282.77	1225.76	26.18	85.86
Evacuation time 45 min				
2300	47.07	211.49	4.40	14.85
5000	102.32	459.75	9.57	32.28
7000	143.24	643.66	13.39	45.20
7500	153.47	689.63	14.35	48.43
10,000	204.63	919.51	19.13	64.57
15,000	306.95	1379.26	28.70	98.85
20,000	409.26	1839.02	38.27	129.14
Evacuation time 60 min				
2300	61.62	282.04	5.80	19.85
5000	133.95	613.13	12.60	43.16
7000	187.53	858.39	17.64	60.42
7500	200.93	919.70	18.90	64.74
10,000	267.91	1226.27	25.20	86.32
15,000	401.86	1839.40	37.80	129.48
20,000	535.81	2452.53	50.41	172.64
Evacuation time 90 min				
2300	90.74	423.24	8.60	29.94
5000	197.27	920.08	18.70	65.08
7000	276.17	1288.12	26.19	91.12
7500	295.90	1380.13	28.06	97.62
10,000	394.53	1840.17	37.41	130.16
15,000	591.80	2760.25	56.11	195.25
20,000	789.06	3680.33	74.82	260.33
Evacuation time 120 min				
2300	119.89	564.55	11.43	40.13
5000	260.63	1227.29	24.85	87.23
7000	364.88	1718.21	34.79	122.13

7500	390.95	1840.93	37.28	130.85
10,000	521.26	2454.58	49.71	174.47
15,000	781.89	3681.87	74.56	261.70
20,000	1042.52	4909.16	99.41	348.93
Evacuation time 180 min				
2300	178.26	847.53	17.15	60.82
5000	387.51	1842.47	37.29	132.22
7000	542.52	2579.45	52.20	185.11
7500	581.27	2763.70	55.93	198.33
10,000	775.02	3684.93	74.57	264.44
15,000	1162.53	5527.40	111.86	396.66
20,000	1550.05	7369.87	149.15	528.88

Assumptions: aerosolisation 90%, height of the plume 30.5 m, deposition velocity 0.3 m/s for 100 µm and 0.002 m/s for 5 µm particles.

Table S5 Committed effective dose and equivalent dose absorbed in the first 10 days by the red bone marrow (RBM) in a victim of a "dirty bomb" attack in the vicinity of the detonation point depending on the activity of the bomb, the distribution of particle sizes (5 μm or 100 μm) and the evacuation time

Activity (Ci)	Fraction of respirable particles 5 μm (%)			
	20	100	20	100
	Effective dose (50 years) (mSv)		RBM Equ. dose (10 days) (mSv)	
Evacuation time 30 min				
2300	372.65	1551.42	36.66	108.99
5000	810.10	3372.66	79.69	236.93
7000	1134.14	4721.73	111.57	331.70
7500	1215.15	5058.99	119.54	355.40
10,000	1620.20	6745.32	159.39	473.86
15,000	2430.30	10,117.98	239.08	710.80
20,000	3240.40	13,490.64	318.78	947.73
Evacuation time 45 min				
2300	534.27	2327.62	53.35	163.92
5000	1161.46	5060.05	115.98	356.34
7000	1626.04	7084.06	162.37	498.88
7500	1742.19	7590.07	173.97	534.51
10,000	2322.92	10,120.09	231.96	712.68
15,000	3484.38	15,180.14	347.95	1069.02
20,000	4645.84	20,240.18	463.93	1425.36
Evacuation time 60 min				
2300	695.96	3104.14	70.10	219.13
5000	1512.96	6748.13	152.40	476.38
7000	2118.14	9447.39	213.35	666.93
7500	2269.44	10,122.20	228.59	714.56
10,000	3025.92	13,496.27	304.79	952.75
15,000	4538.88	20,244.40	457.19	1429.13
20,000	6051.84	26,992.53	609.58	1905.51
Evacuation time 90 min				
2300	1019.54	4658.15	103.78	330.43
5000	2216.38	10,126.42	225.60	718.33
7000	3102.94	14,176.98	315.84	1005.67
7500	3324.58	15,189.63	338.40	1077.50
10,000	4432.77	20,252.83	451.20	1436.67
15,000	6649.15	30,379.25	676.80	2155.00
20,000	8865.53	40,505.67	902.40	2873.33
Evacuation time 120 min				
2300	1343.37	6213.46	137.68	442.89
5000	2920.37	13,507.51	299.31	962.80
7000	4088.52	18,910.52	419.03	1347.92

7500	4380.55	20,261.27	448.96	1444.20
10,000	5840.74	27,015.02	598.61	1925.60
15,000	8761.11	40,522.54	897.92	2888.41
20,000	11,681.48	54,030.05	1197.22	3851.21
Evacuation time 180 min				
2300	1991.81	9327.94	206.18	671.27
5000	4330.03	20,278.14	448.22	1459.28
7000	6062.04	28,389.39	627.51	2042.99
7500	6495.04	30,417.20	672.34	2188.91
10,000	8660.05	40,556.27	896.45	2918.55
15,000	12,990.08	60,834.41	1344.67	4377.83
20,000	17,320.10	81,112.54	1792.90	5837.11

Assumptions: aerosolisation 90%, plume size as in the Oslo bombing (radius and height 40 m), deposition velocity 0.3 m/s for 100 µm and 0.002 m/s for 5 µm particles.

Table S6 Committed effective dose and equivalent dose absorbed within the first 10 days by the red bone marrow (RBM) in a victim of a “dirty bomb” attack in a subway depending on the evacuation time and the distribution of particle sizes (5 µm or 100 µm)

Evacuation time (min)	Fraction of respirable particles 5 µm (%)					
	0	10	20	0	10	20
	Effective dose (50 years) (mSv)			RBM Equivalent dose (10 days) (mSv)		
15	1061.33	13,924.44	26,787.54	178.91	1059.87	1940.84
20	1101.42	18,284.79	35,468.16	214.00	1392.81	2570.89
25	1141.51	22,645.75	44,150.00	250.55	1726.28	3202.01
30	1181.59	27,007.31	52,833.03	286.37	2060.29	3834.21
45	1301.85	27,127.57	52,953.29	393.83	2167.75	3941.67
60	1422.10	27,247.82	53,073.54	501.30	2275.21	4049.13
90	1662.62	27,488.34	53,314.06	716.22	2490.14	4264.06
105	1782.87	27,608.59	53,434.31	823.69	2597.60	4371.52
120	1903.13	27,728.85	53,554.57	931.15	2705.07	4478.99
150	2143.64	27,969.36	53,795.08	1146.08	2919.99	4693.91
180	2384.16	28,209.88	54,035.60	1361.00	3134.92	4908.84

Assumptions: cesium-137 activity 2300 Ci, aerosolisation 90%, subway length 115 m, width 2.90 m, height 3.60 m (Munich subway type C2), deposition velocity 0.3 m/s for 100 µm and 0.002 m/s for 5 µm particles.
